# POMS enhances open spectral library search for identification of modified peptides

**DOI:** 10.1186/s12859-026-06672-0

**Published:** 2026-09-21

**Authors:** Younghee Seo, Eunok Paek, Seungjin Na

**Affiliations:** 1 Center for Target-to-Therapeutics Research, Korea Basic Science Institute, Cheongju, 28119 Republic of Korea; 2 Department of Computer Science, Hanyang University, Seoul, 04763 Republic of Korea; 3 Department of Artificial Intelligence, Hanyang University, Seoul, 04763 Republic of Korea; 4 Convergent Analytical Science, University of Science and Technology, Daejeon, 34113 Republic of Korea

**Keywords:** Proteomics, Tandem mass spectrometry, Post-translational modifications, Open search, Spectral-library search

## Abstract

**Background:**

Peptide identification from tandem mass spectrometry (MS/MS) data is a central task in proteomics. Spectral library searching combined with open modification search (OMS) has emerged as an effective strategy for identifying peptides carrying unexpected post-translational modifications (PTMs). However, existing methods do not adequately account for modification-induced fragment-ion shifts during candidate selection, leading to missed identifications when mass shifts distort spectral similarity.

**Results:**

We present POMS, a modification-aware framework for open spectral library search that improves candidate retrieval by integrating complementary spectral representations with sequence-derived theoretical fragment features. These representations compensate for modification-induced fragment-ion shifts and improve alignment between query and library spectra, increasing robustness to modification site variability. Benchmarking on large-scale human MS/MS datasets showed that POMS yielded up to ~ 8% more peptide identifications during the open search phase than conventional methods. Cross-validation with independent database search engines further demonstrated a > 5% increase in consistent peptide identifications. Notably, POMS substantially improved the identification of peptides carrying C-terminal modifications, for which conventional candidate retrieval methods are particularly susceptible to fragment-ion shifts.

**Conclusions:**

POMS improves the sensitivity and reliability of modification-tolerant spectral library searching by incorporating modification-aware candidate retrieval while preserving computational efficiency. The framework provides a practical solution for large-scale PTM discovery and is readily applicable to existing spectral library search workflows. POMS is publicly available under the CC BY-NC-SA 4.0 license at https://github.com/icp-kbsi/POMS.

**Supplementary Information:**

The online version contains supplementary material available at https://doi.org/10.1186/s12859-026-06672-0.

## Background

 Tandem mass spectrometry (MS/MS)-based peptide analysis is a fundamental yet complex task in proteomics, essential for identifying and characterizing proteins within biological samples [1, 2]. To enhance the interpretation of MS/MS data, numerous computational approaches have been proposed, among which spectral library search [3–7], by comparing query spectra directly with previously validated reference spectra in spectral libraries, achieves higher sensitivity and computational efficiency than traditional sequence database search [8–11]. Unlike sequence database search, which compares experimental spectra against theoretical spectra generated from protein sequences, spectral library search exploits experimentally observed fragmentation patterns, enabling more accurate spectral matching when representative reference spectra are available.

A major challenge in proteomics remains the identification of post-translational modifications (PTMs), as conventional methods are generally limited to identifying only pre-specified modifications [12, 13]. Standard approaches typically require users to define a limited set of expected modifications, thereby restricting the search space and precluding the discovery of unexpected or novel modifications. To address these limitations, Open Modification Search (OMS) has emerged as a powerful strategy for identifying peptides carrying a broad range of modifications without prior specification [14–18]. OMS has been successfully implemented in both sequence database search and spectral library search. In both settings, OMS employs a wide precursor mass tolerance, typically up to several hundred Daltons (e.g., 500 Da), which dramatically increases the number of candidates and consequently the computational burden. Therefore, efficient candidate retrieval has become a key component of scalable OMS methods. To improve scalability, database-based OMS methods employ various indexing strategies to efficiently retrieve candidate peptide sequences. Similarly, in spectral library-based OMS, ANN-SoLo employs an Approximate Nearest Neighbor (ANN)-based indexing strategy to prioritize candidate spectra from the spectral library, thereby significantly reducing pairwise spectral comparisons [19, 20]. Through feature hashing, ANN-SoLo converts m/z and intensity values into vector representations that enable efficient similarity searches in high-dimensional space. Unlike the subsequent spectrum scoring step, which explicitly aligns fragment ions while accounting for precursor mass shifts, ANN-based candidate retrieval relies solely on vector similarity before detailed spectral comparison. Consequently, modification-induced fragment-ion shifts can distort vector representations and reduce the likelihood that the correct library spectrum is retrieved for subsequent scoring. Although ANN-SoLo greatly improves computational efficiency, its candidate retrieval strategy does not explicitly account for these fragment-ion shifts, thereby limiting its sensitivity for identifying modified peptides.

In this study, we propose POMS, a modification-aware framework for open spectral library search that integrates complementary spectral representations [21, 22] and sequence-derived theoretical fragment features [23, 24] to improve candidate retrieval for modified peptides. The complementary spectral representation models shifted fragment-ion relationships between query and library spectra, mitigating vector-space distortion caused by modification-induced mass shifts. In parallel, sequence-derived theoretical fragment features suppress the influence of noise peaks and provide sequence-informed guidance during candidate retrieval. Together, these complementary representations refine candidate retrieval and improve robustness to modification site variability. In particular, POMS substantially improves the identification of peptides carrying C-terminal modifications, for which long y-ion series undergo extensive mass shifts that severely distort conventional ANN vector representations.

Benchmarking on large-scale human MS/MS datasets demonstrated that POMS identifies substantially more modified peptides during open spectral library search than ANN-SoLo. Cross-validation with independent sequence database search engines further confirmed the reliability of these additional identifications, demonstrating that the peptides uniquely recovered by POMS are supported by an independent search paradigm rather than arising from ANN-based retrieval artifacts. In particular, POMS shows marked improvements in identifying peptides carrying C-terminal modifications, highlighting the effectiveness of the proposed modification-aware candidate retrieval strategy.

## Materials and methods

### Overview of ANN-SoLo

**Fig. 1:**
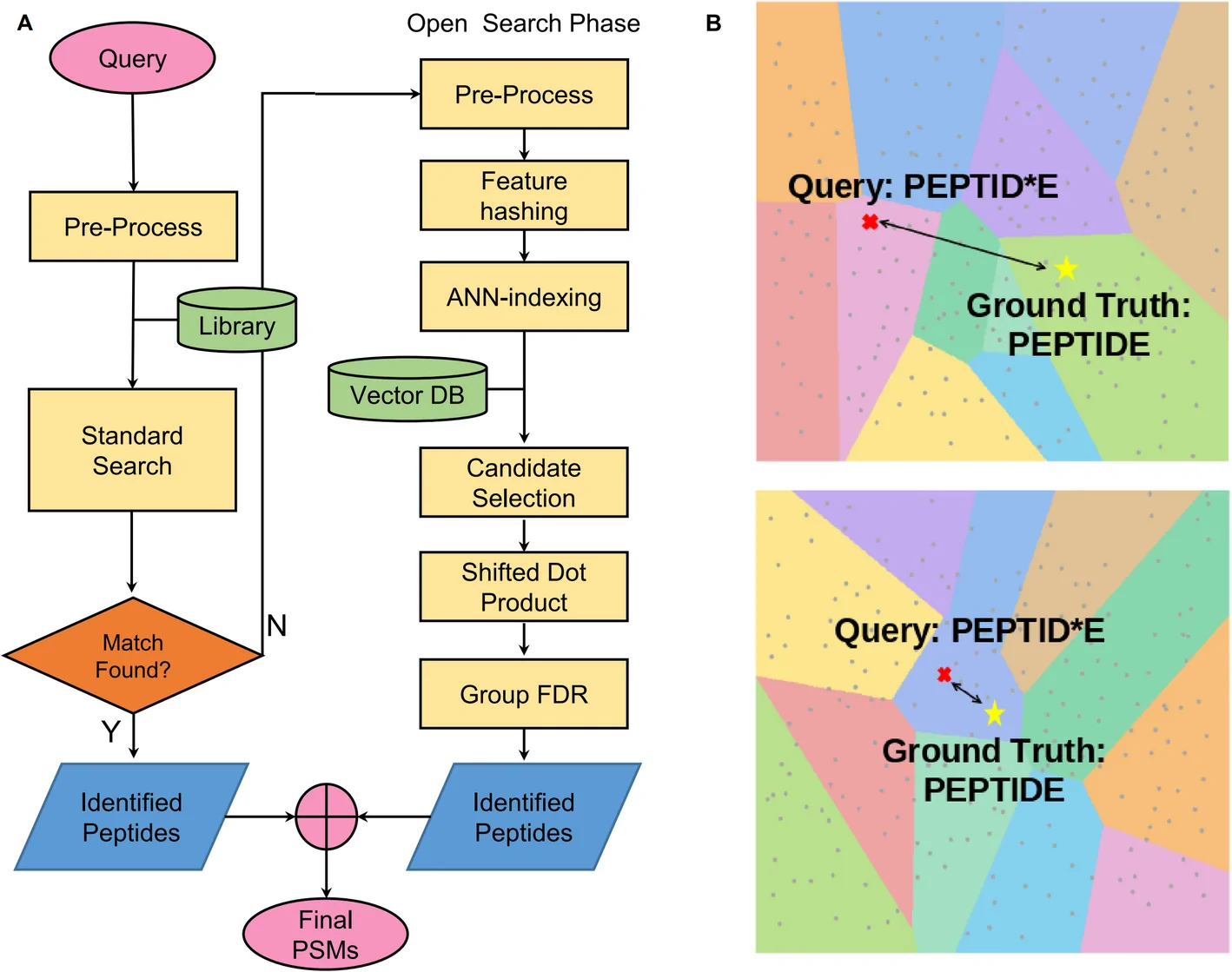
Overview of the ANN-SoLo workflow and its limitation in open modification search. **A** Two-stage search strategy of ANN-SoLo. A standard search first identifies unmodified peptides or peptides with predefined modifications, followed by an ANN-based open search applied to the remaining unidentified spectra. **B** Illustration of the candidate retrieval limitation. Because ANN-SoLo clusters spectra using intensity-derived spectral vectors without explicitly modeling modification-induced fragment-ion shifts, modified query spectra may fall outside the retrieval radius of their corresponding unmodified library centroids, resulting in missed identifications. (Note: Fig. 1B is a conceptual schematic illustrating the candidate retrieval principle and does not represent actual relative positions in the high-dimensional vector space)

ANN-SoLo is a spectral library search engine designed to facilitate efficient OMS by integrating ANN indexing for candidate retrieval. To enhance search efficiency, ANN-SoLo employs a two-stage search strategy. Initially, it performs a standard search to identify unmodified peptides or those with pre-defined modifications. For spectra that remain unidentified, ANN-SoLo proceeds to an open search, broadening the precursor mass tolerance and expanding the search space to identify potential modifications (Fig. 1A).

For candidate retrieval, each spectrum in the spectral library is converted into a high-dimensional vector representation using feature hashing. Fragment peaks are binned according to their m/z values, and corresponding intensities are mapped into a fixed-length vector space through a hashing function. This process produces compact hash vectors that approximate spectral similarity while enabling efficient similarity searches in high-dimensional space. To accelerate this process, ANN-SoLo employs the Facebook AI Similarity Search (Faiss) library to construct an ANN index over the hash vectors [25]. During the search, a query spectrum is converted into the same vector representation to query the ANN index, which returns a prioritized set of candidate spectra. This strategy substantially reduces the number of pairwise spectral comparisons required during OMS. To evaluate candidates, a shifted dot product is applied, which allows fragment-ion matches under a constant mass offset equal to the precursor mass difference, enabling identification of modified peptides. Finally, false discovery rate (FDR) is estimated using a group-based strategy in which peptide-spectrum matches (PSMs) are categorized by precursor mass differences. A minimum group size of 20 is required; groups falling below this threshold are merged into a combined category for FDR estimation.

### Complementary peak generation

**Fig. 2:**
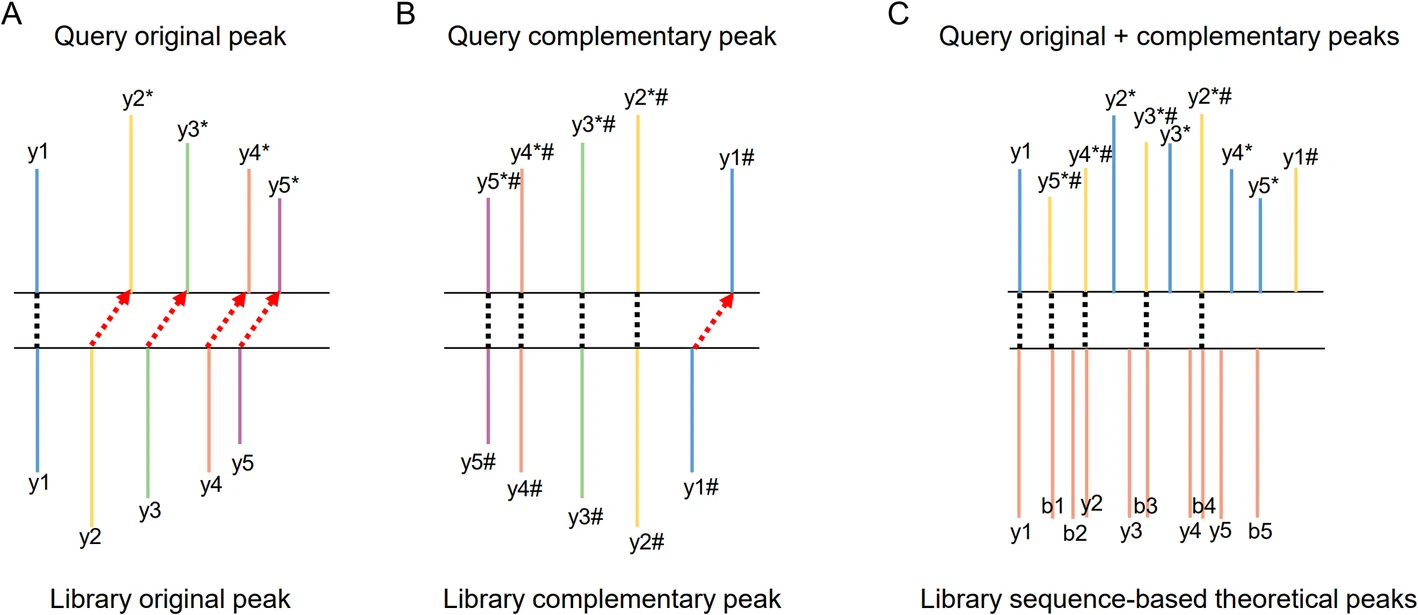
Three strategies for matching an experimental query spectrum (top) with a library spectrum (bottom). **A** Original peak matching, where fragment ions are directly compared between the query and library spectra. **B** Complementary peak matching, in which fragment ions are transformed to their complementary positions to align modification-shifted peaks in the query with their corresponding unmodified fragment ions in the library. **C** Sequence-informed representation, where the query spectrum is represented by combining original and complementary peaks, while the library spectrum is represented using a sequence-derived theoretical spectrum to capture peptide backbone similarity. In all panels, * denotes modification-shifted peaks, and # indicates the corresponding complementary peaks

While ANN-SoLo significantly improves computational efficiency, the ANN-based candidate retrieval step relies solely on hashed spectral representations derived from fragment m/z and intensity values and does not explicitly model modification-induced fragment-ion shifts. Consequently, modification mass shifts can distort the vector-space similarity between modified and unmodified spectra, potentially excluding true matches from the candidate set prior to scoring (Fig. 1B). This limitation is particularly acute for C-terminal modifications, where y-ions—typically the most abundant fragments in collision-induced dissociation (CID) and higher-energy collisional dissociation (HCD)—are systematically shifted, thereby diminishing the spectral similarity between modified query spectra and their unmodified library counterparts as illustrated in Fig. 2A.

To overcome this limitation in ANN-SoLo, we implemented a complementary peak generation strategy that enables modification-shifted fragment peaks in a query spectrum to align with their corresponding unmodified fragment peaks in the reference library spectrum (Fig. 2B). Specifically, for each observed fragment ion with mass $\:{m}_{obs}$, its complementary fragment mass $\:{m}_{comp}$ is calculated based on the precursor mass $\:{M}_{precursor}$:

$$\:{m}_{comp}={M}_{precursor}+2{H}^{+}-{m}_{obs}$$

where $\:{H}^{+}$ denotes the proton mass. This relationship reflects the complementary nature of b- and y-ion pairs in peptide fragmentation. For example, a peak treated as a b-ion can be used to infer its corresponding y-ion position, and vice versa. If a modification occurs near the C-terminus, the resulting mass shift propagates through the y-ion series, causing modified y-ions in the query spectrum to deviate from their corresponding unmodified library peaks. Through complementary transformation, these modified y-ions are converted into complementary b-ion positions that correspond to unmodified fragment positions, which remain aligned with the unmodified fragment peaks in the reference spectrum. The newly generated complementary fragment peak retains the intensity of its corresponding original fragment peak. Consequently, the complementary representation restores spectral correspondence between modified query spectra and their unmodified library counterparts during candidate retrieval.

### Sequence-derived theoretical fragment ions

To bolster candidate retrieval robustness, we incorporated sequence-derived information into spectral comparison. This approach leverages the core principles of traditional sequence database searching, where peptide identification is guided by theoretical fragment-ion positions. Unlike experimental spectra, which often contain stochastic noise or electronic interference, sequence-derived theoretical features provide a deterministic representation of the peptide backbone structure. By focusing exclusively on theoretical fragment-ion m/z values, these features reduce the influence of non-peptidic noise and stochastic spectral variation, thereby facilitating the capture of fundamental peptide backbone similarity.

Theoretical fragment ions derived from the library peptide sequences were compared with the combined representation of original and complementary peaks from the query spectrum (Fig. 2C). This strategy enables prioritization of candidates with high peptide backbone similarity during ANN-based retrieval, even when raw spectral similarity is degraded due to incomplete or noisy fragmentation.

### POMS spectral embedding

By integrating complementary spectral representations and sequence-derived theoretical fragment features into the spectrum vector through a concatenation-based embedding strategy, POMS constructs an augmented embedding designed to improve candidate retrieval under modification-induced spectral distortion.

The embedding architecture generates a dual-component vector for both library and query spectra to mitigate vector-space discrepancies introduced by modifications. For each library spectrum, the first component (800 dimensions) consists of theoretical b- and y-ion m/z positions derived from the peptide sequence, encoded as a binary vector in which theoretical fragment-ion bins are assigned a value of 1 and all other bins are set to 0, thereby providing a deterministic backbone-level template. This component is concatenated with the second component, which corresponds to the hashed representation of the library spectrum. For the query spectrum, where the peptide sequence is unknown, the first component is constructed from the combined representation of the experimental spectrum and its complementary spectrum as described in Fig. 2C, serving as a surrogate backbone representation. Specifically, the complementary spectrum is generated as described previously, and its binned intensities are summed element-wise with those of the original experimental spectrum. The resulting combined vector constitutes the first component of the query embedding. This component is then concatenated with the hashed experimental query spectrum to form the final query embedding.

This augmented representation enables ANN-based retrieval to capture both backbone-level structural similarity and experimental peak-level similarity. Consequently, modified query spectra remain closer to their corresponding unmodified library spectra in the vector space used for candidate retrieval, even under substantial modification-induced mass shifts. Using the concatenated embeddings, a new ANN index was constructed using the Faiss library following the retrieval procedure established by ANN-SoLo. The downstream scoring stage, including shifted dot product evaluation and FDR estimation, remains unchanged to ensure that observed performance gains arise solely from improvements in candidate retrieval robustness.

### Parallel multi-representation search strategy

**Fig. 3:**
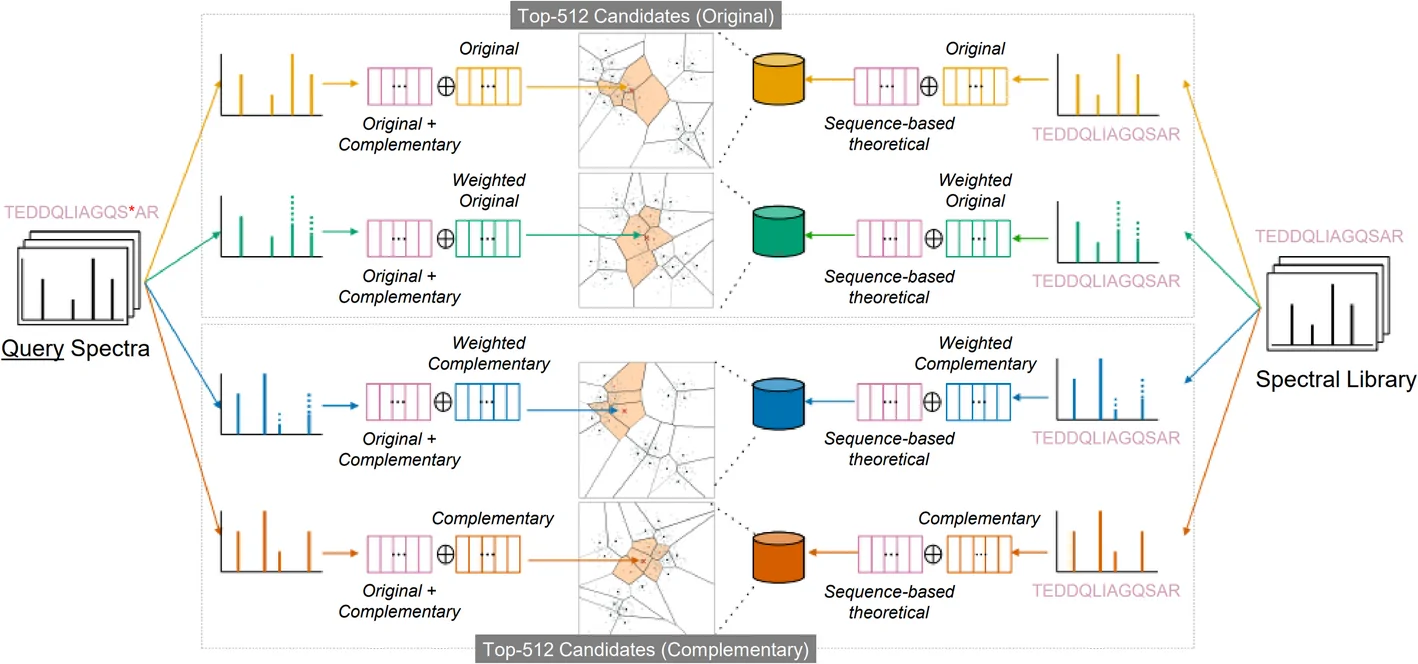
Overview of the POMS framework. The workflow integrates complementary spectral representations and sequence-derived theoretical fragment features for candidate retrieval. Prior to the search, a spectral vector database is constructed, in which library spectra are transformed into four representations (original, weighted original, weighted complementary, and complementary), and sequence-derived theoretical spectra are generated from peptide sequences. For each experimental query spectrum, the same four spectral representations are generated. Following the dual-embedding strategy, four parallel searches are performed using concatenated vectors. Specifically, each concatenated vector combines a sequence embedding with one of the four spectral embeddings: for the query, the sequence embedding is constructed by combining original and complementary peaks, whereas for the library, it is derived from the theoretical spectrum. Based on vector similarity, the top 512 candidates are retrieved from the original and weighted original representations, and an additional 512 candidates are retrieved from the complementary and weighted complementary representations, resulting in a total of 1,024 candidates per query

To enhance sensitivity to modification site–dependent spectral distortion, POMS performs four parallel candidate retrieval searches, each based on a distinct representation of the spectral library (Fig. 3). This design reflects the observation that modification-induced mass shifts affect different regions of the fragment-ion series depending on the modification location. The spectral library is represented by four distinct models:

1. Standard Hashing: Uses the original spectral intensities.
2. High-m/z Down-weighted Hashing: Reduces the intensities of high-m/z peaks by 50% to mitigate the dominance of modification-shifted ions. For the *k*-th peak with m/z value $\:{m}_{k}$ and intensity $\:{i}_{k}$, the adjusted intensity $\:{i^{\prime\:}}_{k}$ is defined as:

$$\:{i^{\prime\:}}_{k}=0.5\times\:{i}_{k},\:\:if\:{m}_{k}\ge\:{M}_{precursor}/2$$

$$\:{i^{\prime\:}}_{k}={i}_{k},\:\:\:\:\:\:\:\:\:\:\:\:\:\:otherwise$$

3. Complementary Hashing with Down-weighting: Incorporates complementary fragment ions together with high-m/z intensity down-weighting.
4. Full Complementary Hashing: Uses a complementary-spectrum representation without intensity down-weighting.

For each query spectrum, four corresponding representations are generated. Each query representation is searched independently against the corresponding library representation using the ANN index. The candidate spectra retrieved from all four searches are then merged into a unified candidate set prior to scoring.

This multi-representation strategy accounts for the non-uniform spectral distortion patterns arising from N-terminal, internal, or C-terminal modifications. As illustrated by the spectral examples in Supplementary Fig. S1, the most effective representation depends on the modification location. The standard hashing representation performs best for N-terminal modifications (Fig. S1A), whereas high-m/z down-weighted or complementary down-weighted representations are more effective for modifications located in the middle of the peptide (Fig. S1B). For C-terminal modifications, the full complementary representation most effectively restores spectral similarity (Fig. S1C). By diversifying the spectral representations, POMS increases the likelihood that at least one spectral representation preserves sufficient similarity between modified query spectra and their corresponding library spectra during candidate retrieval. All retrieved candidates are subsequently evaluated using the same shifted dot product scoring and FDR estimation procedures as implemented in ANN-SoLo.

### MS/MS dataset and analysis

Three large-scale MS/MS datasets were downloaded from the ProteomeXchange repository: HEK293 (PXD001468), human liver (PXD000561) and phosphoproteome (PXD004452). The HEK293 dataset, consisting of 1,121,149 spectra across 24 runs, was acquired using a Q-Exactive instrument (Thermo Scientific) with HCD [14]. The human liver dataset comprised 392,674 spectra from 24 runs, obtained via an Orbitrap Elite instrument (Thermo Scientific) with HCD [26]. The phosphoproteome dataset, phosphorylation enrichment of HeLa cell, comprised 513,720 spectra, acquired using a Q-Exactive HF instrument (Thermo Scientific) with HCD [27]. Detailed specifications of these datasets are available in their original publications. For the spectral library, we used 2,140,865 high-resolution human peptide HCD spectra from the MassIVE-KB repository.

Both datasets were searched against the MassIVE-KB library to compare the performance of POMS and ANN-SoLo. The search configurations were kept consistent across both tools: the fragment mass tolerance was set to ± 0.02 m/z, and the precursor mass tolerance was set to ± 20 ppm for the first-stage standard search and expanded to ± 500 Da for the second-stage open search. The search results were validated using a target–decoy strategy [28] and the final identifications were obtained at 1% FDR.

To benchmark POMS, we performed independent open database searches using pFind3 (v3.2.0) [16] and MSFragger (v3.5) [15] via FragPipe (v18.0). The search parameters were as follows: enzyme = trypsin, number of enzymatic termini = 2, missed cleavages ≤ 2, precursor mass tolerance = ± 20 ppm, fragment mass tolerance = ± 20 ppm, variable modifications = Oxidation (Met) and Acetyl (protein N-terminus), fixed modification = Carbamidomethyl (Cys), maximum number of variable modifications per peptide = 3, and maximum precursor delta mass = 500 Da. The reference proteome was obtained from the UniProtKB/Swiss-Prot human database (Sep 20, 2022), and all searches employed target-decoy competition to ensure a 1% FDR.

## Results

### Improved identification of modified peptides compared with ANN-SoLo

We evaluated the performance of POMS using two human MS/MS datasets from HEK293 cells and liver tissue. The identification results are summarized in Table 1. Consistent with the original ANN-SoLo framework, a two-stage search strategy was employed: an initial standard search for unmodified or pre-defined modified peptides, followed by an open search for the remaining unidentified spectra. POMS retains this search framework, and therefore the identification of unmodified peptides remains identical between the two methods.

**Table 1:**
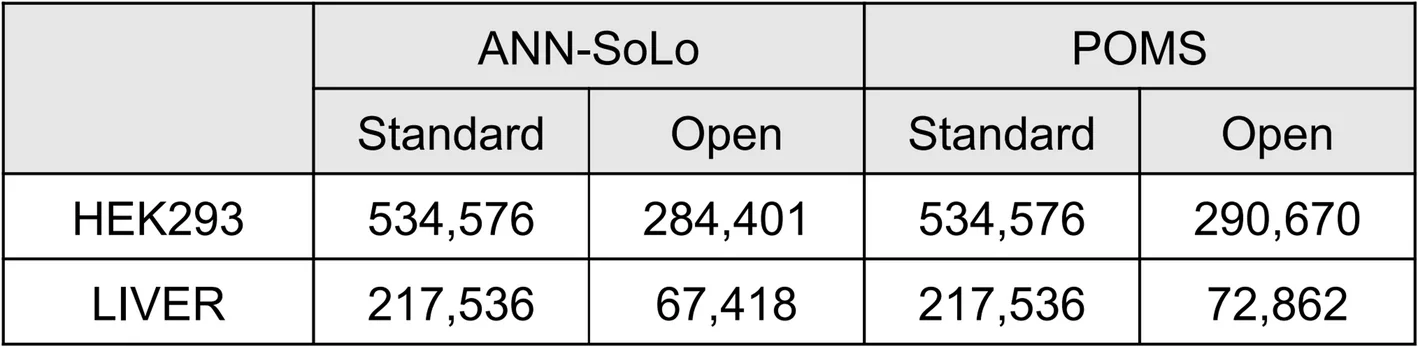
Number of PSMs identified during the standard search and open search stages by ANN-SoLo and POMS

In contrast, POMS improved the identification of modified peptides during the open search stage. Specifically, POMS yielded 2.2% and 8.1% increases in modified peptide identifications for the HEK293 and liver datasets, respectively, relative to ANN-SoLo. Because POMS modifies only the candidate retrieval stage while preserving the scoring and FDR procedures of ANN-SoLo, the observed gains can be attributed directly to improved candidate retrieval. This result underscores the ability of the proposed complementary and sequence-informed representations to recover modified spectra that are otherwise missed during ANN-based candidate retrieval. To clarify the contributions of the proposed features, ablation analysis demonstrated that integrating both complementary peaks and sequence embeddings, as implemented in the full POMS model, provided robust performance improvements across different proteomic datasets, whereas the relative benefit of each individual feature varied across datasets (Supplementary Table S1).

**Fig. 4:**
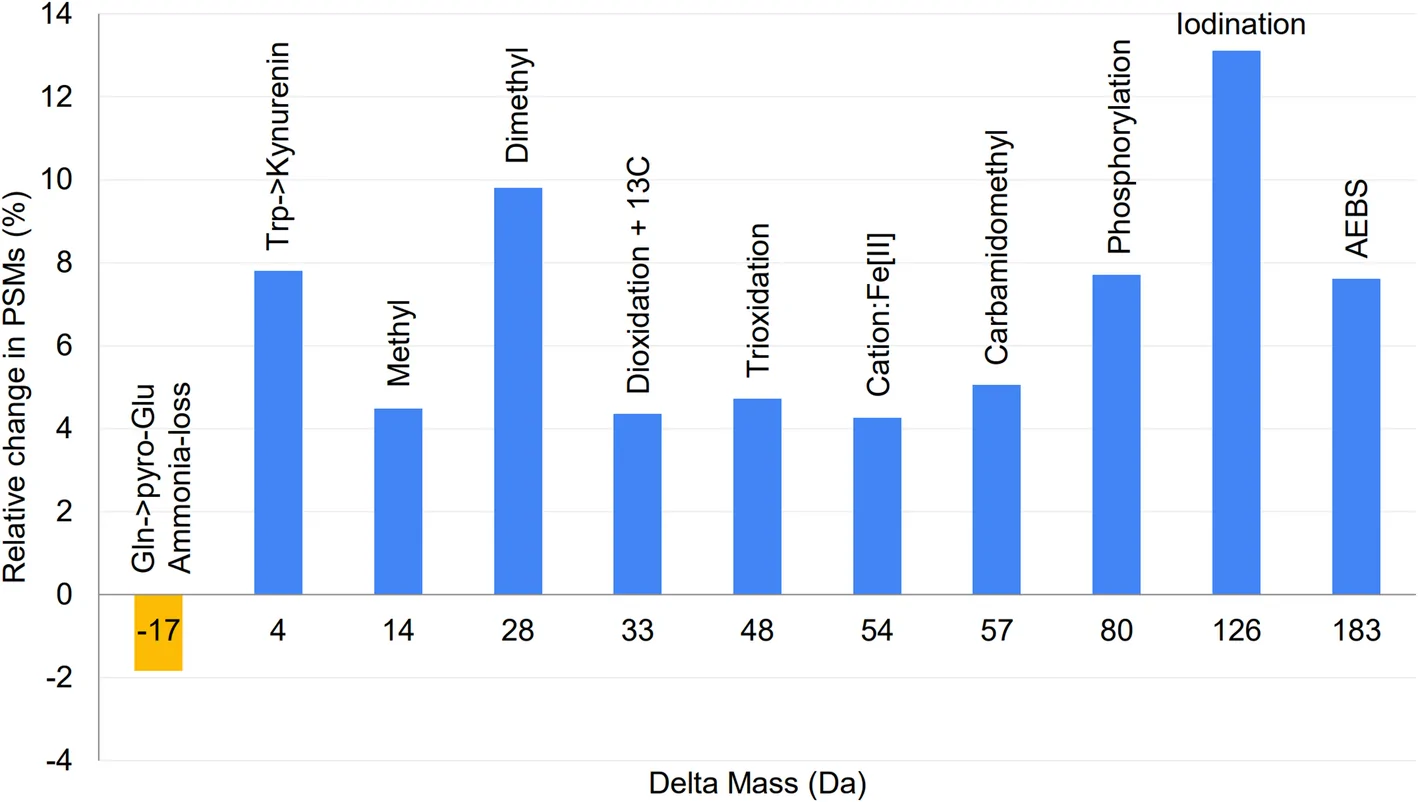
Comparison of modification frequency distributions identified from HEK293 dataset by POMS and ANN-SoLo. The bar chart displays modification masses for which each method identified a higher number of PSMs. Upward blue bars represent modification masses where POMS identified more PSMs than ANN-SoLo, whereas downward orange bars indicate masses where ANN-SoLo identified more PSMs. Only modification masses supported by at least 300 PSMs were included to focus on reliably observed modification events. The corresponding modification annotation for each delta mass is indicated for each bar

To further characterize these improvements, we analyzed the distribution of modification types detected by each method. To focus on reliably observed modifications, the analysis was restricted to modification delta masses detected by at least 300 PSMs, while low-frequency delta masses were excluded because sporadic mass shifts in open searches are often associated with random noise or experimental artifacts rather than recurrent modification events. As shown in Fig. 4, POMS outperformed ANN-SoLo across a wide range of high-frequency modification types. The upper bars indicate modification masses for which POMS identified more PSMs than ANN-SoLo, suggesting that these modification types were frequently missed by ANN-SoLo but successfully recovered by POMS. Notably, the modifications preferentially recovered by POMS correspond to modification mass shifts that are frequently observed in the datasets. In contrast, ANN-SoLo showed higher identification counts in only one delta mass. These results demonstrate that POMS consistently improves the detection of prevalent and biologically relevant PTM types while maintaining reliable identification performance.

To further evaluate whether the advantage of POMS extends to modification-rich data, we analyzed an additional phosphoproteomics dataset enriched for phosphorylated peptides. POMS improved the identification of phosphorylated peptides compared with ANN-SoLo (Supplementary Fig. S2). Notably, even greater improvements were observed for peptides carrying multiple phosphorylation sites. These results demonstrate that the advantage of POMS becomes more pronounced for heavily modified peptides, where multiple modification-induced fragment-ion shifts can substantially distort spectral similarity and hinder conventional ANN-based candidate retrieval.

### Validation and characterization of POMS identifications

**Fig. 5:**
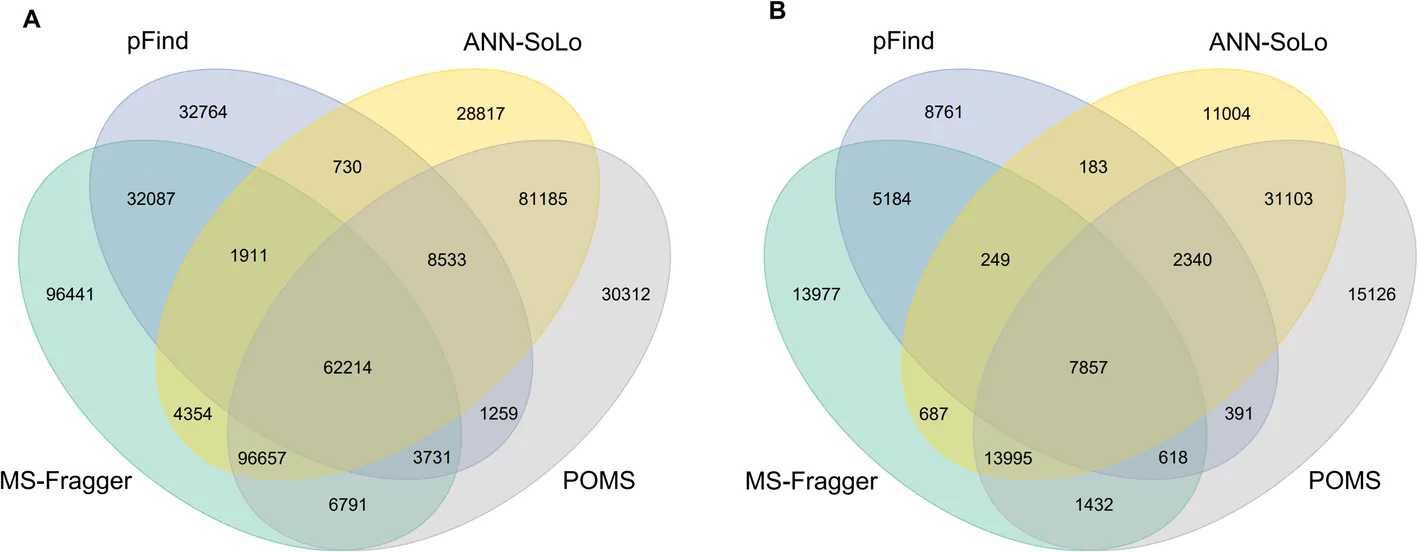
Validation of PSMs identified by ANN-SoLo and POMS using independent database search methods. Venn diagrams show the overlap between PSMs identified by ANN-SoLo or POMS during open search and those identified by pFind3 and MSFragger for (**A**) the HEK293 dataset and (**B**) the human liver dataset. Only spectra identified by ANN-SoLo or POMS were considered in this comparison. Overlap was defined when the same peptide sequence was assigned to the same query spectrum across methods. This comparison assesses the reliability of identifications obtained by ANN-based spectral library searching

To validate the reliability of the modified peptide identifications recovered by POMS, we performed independent open database searches using pFind3 and MSFragger. The overlap between PSMs identified by ANN-SoLo or POMS and those identified by independent database search methods is illustrated in Fig. 5. In this comparison, only spectra identified by ANN-SoLo or POMS were considered, and identifications were defined as overlapping when the same peptide sequence was assigned to the same query spectrum across methods. The comparison revealed that modified PSMs uniquely identified by POMS exhibit higher agreement with pFind3 and MSFragger than those uniquely identified by ANN-SoLo. This increased concordance with independent database search results indicates that the additional identifications produced by POMS are of high reliability. For completeness, the global overlap of all identified PSMs across the four search methods is provided in Supplementary Fig. S3.

To elucidate the performance gains achieved by POMS, we analyzed the distribution of modification sites along the peptide sequence. Because ANN-SoLo and POMS do not explicitly localize modification sites, the modification positions were determined using the results from database-based open searches (pFind3 and MSFragger), which provide explicit modification localization. Specifically, only PSMs with sequence agreement between POMS (or ANN-SoLo) and pFind3 (or MSFragger) were considered, and the modification positions assigned by pFind3 and MSFragger were used as the reference localization for position-specific analysis.

**Fig. 6:**
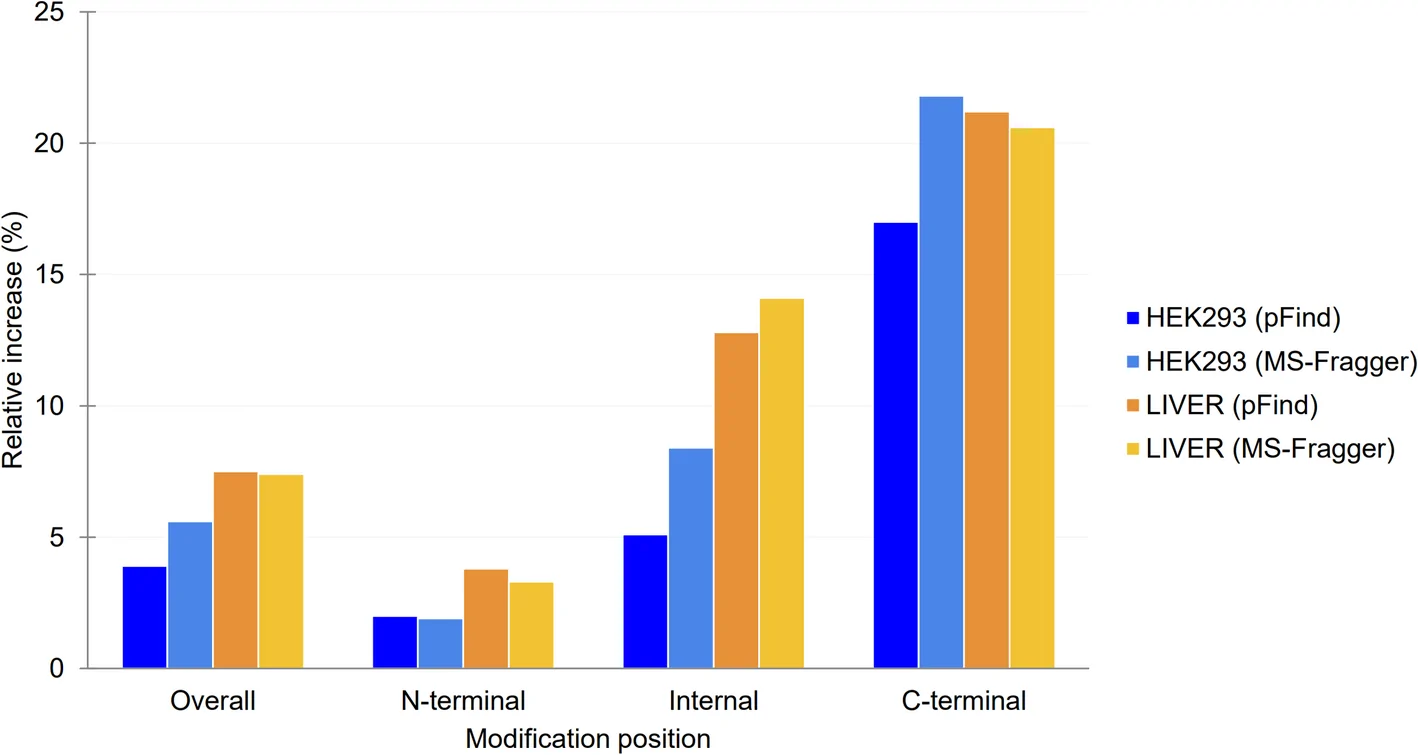
Improvement in modified peptide identification by POMS across modification position. Bar plots show the relative increase in correctly identified PSMs by POMS compared with ANN-SoLo for N-terminal, internal, and C-terminal modifications in the HEK293 and liver datasets. Modification positions were determined based on localization results from database open search methods

The identified PSMs were then categorized according to the relative position of the modification within the peptide sequence: N-terminal, internal (middle), and C-terminal. To simplify the analysis, peptides containing multiple PTMs were excluded from this analysis. The most pronounced improvement was observed for C-terminal modifications. In the HEK293 dataset, POMS recovered approximately 1,000 additional C-terminal modified PSMs compared with ANN-SoLo, and a similar trend was observed in the liver dataset. The relative improvement across modification positions is summarized in Fig. 6, which clearly highlights the disproportionate gain at the C-terminus.

This pattern reflects the fragmentation characteristics of HCD spectra, in which y-ions are typically dominant. Modifications located near the C-terminus introduce systematic mass shifts across a large portion of the y-ion series, substantially reducing spectral similarity to unmodified library spectra. As a result, such spectra are often excluded during candidate retrieval in ANN-SoLo, which relies on intensity-based spectral hashing. By incorporating complementary spectral representations and sequence-informed features during candidate retrieval, POMS mitigates this limitation and enables effective retrieval of spectra containing C-terminal modifications. These results demonstrate that POMS successfully addresses a key limitation of ANN-based spectral library searching by substantially improving the identification of peptides with C-terminal modifications.

### Improved alignment between vector similarity and spectral scoring

To further examine why POMS improves modified peptide identification, we analyzed the relationship between vector similarity used during candidate retrieval and the shifted dot product score used for final spectrum matching. Ideally, the retrieval metric should correlate well with the final scoring function so that spectra with high true similarity are preferentially retrieved during the ANN search.

**Fig. 7:**
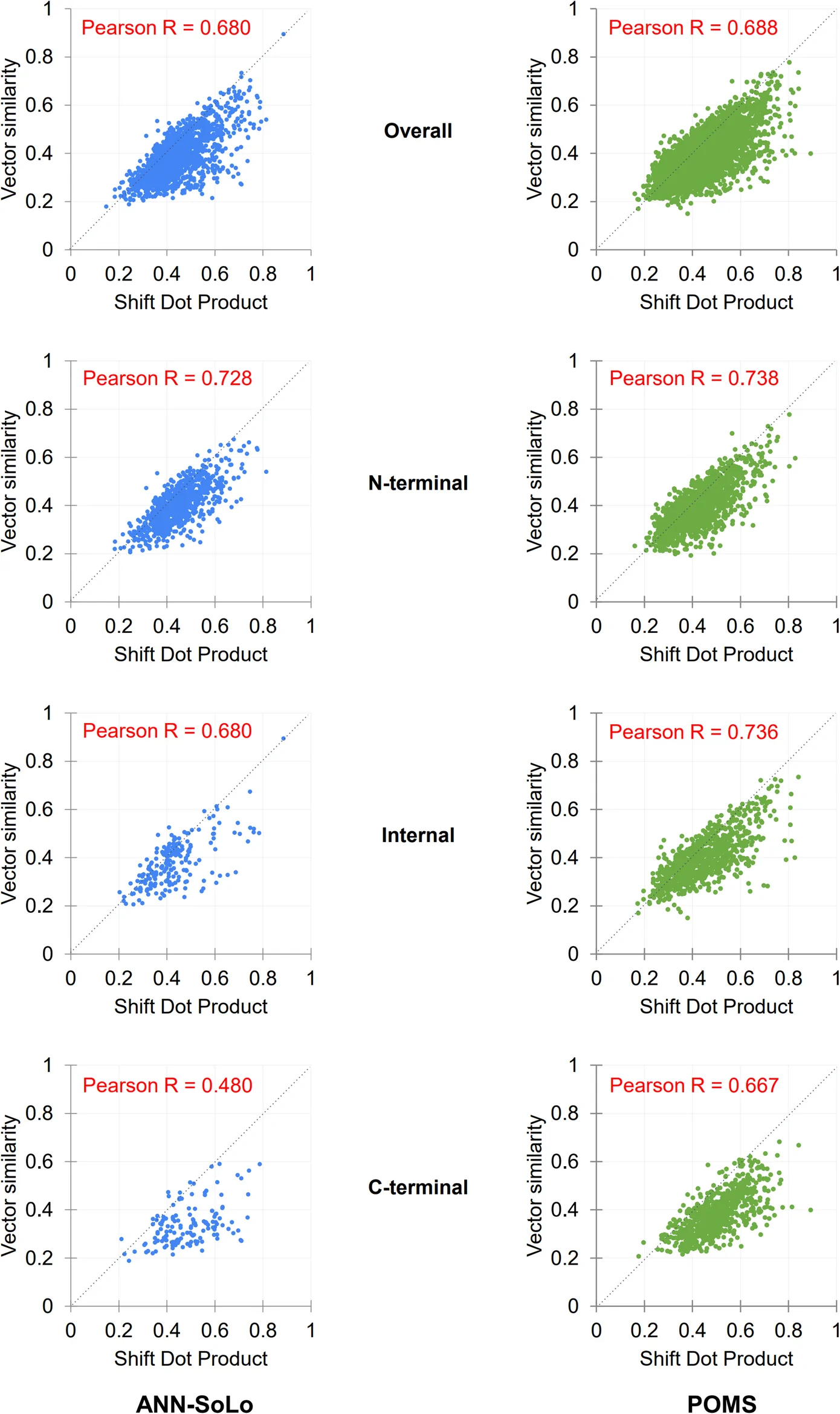
Correlation between vector similarity and shifted dot product scores. Scatter plots compare the relationship between vector inner-product similarity used for candidate retrieval and shifted dot product scores used for final spectrum matching. Results for ANN-SoLo are shown on the left and POMS on the right. Panels from top to bottom show correlations for all spectra, N-terminal, internal, and C-terminal modifications, respectively. POMS exhibits a stronger correlation between retrieval similarity and spectral scoring, particularly for C-terminal modifications

We compared the inner product between spectral vectors with the shifted dot product scores for matched peptide-spectrum pairs. As shown in Fig. 7, ANN-SoLo exhibits moderate correlation when modifications are located near the N-terminus but shows substantially reduced correlation for C-terminal modifications. This behavior reflects the distortion introduced by systematic shifts of the dominant y-ion series, which disrupt the vector-space similarity used for candidate retrieval.

In contrast, POMS maintains a substantially stronger correlation between vector similarity and shifted dot product scores across all modification positions, with the largest improvement observed for C-terminal modifications. This result demonstrates that the augmented spectral representation used in POMS better preserves the underlying spectral similarity that ultimately determines spectrum matching. Consequently, POMS is more likely to retrieve correct candidates during the ANN search stage, particularly for spectra containing modification-induced fragment-ion shifts.

Furthermore, to directly quantify the improvement in candidate retrieval, we evaluated the Top-*k* retrieval recall using ground-truth PSMs identified from the HEK293 and human liver datasets by pFind. Top-*k* recall was defined as the proportion of ground-truth PSMs for which the correct peptide was represented among the Top-*k* candidates. As shown in Supplementary Fig. S4, POMS consistently achieved higher recall than ANN-SoLo across all Top-*k* thresholds, including a 5.69-percentage-point improvement at Top-100. These results provide direct evidence that the enhanced spectral representation of POMS increases the probability of retrieving the correct peptide during the candidate retrieval stage, thereby allowing more correct candidates to proceed to the subsequent fine-scoring stage.

### Runtime performance of POMS

We evaluated the computational efficiency of POMS relative to ANN-SoLo using the HEK293 and liver datasets. Detailed index architectures, vector dimensionalities, and search parameters for both ANN-SoLo and POMS are summarized in Supplementary Table S2. To accommodate the expanded 1,600-dimensional concatenated vector space and four independent sub-indices used in POMS, the ANN search parameters (e.g., nlist and nprobe) were adjusted to maintain practical search runtimes. All runtime evaluations were performed on a server running Ubuntu 24.04.3 LTS (kernel 6.8.0-117-generic), equipped with dual-socket Intel Xeon Platinum 8562Y+ CPUs (64 physical cores, 128 threads) and 1,024 GB of DDR5 RAM. The searches were performed in CPU-only mode using Python 3.7.16, Pyteomics (v4.7.5), and FAISS-CPU (v1.7.4), with 128 OpenMP threads.

The total search time included all processing steps, such as spectral loading, index querying, and open search evaluation. For the HEK293 dataset, POMS recorded a total processing time of 18,451 s, compared with 12,320 s for ANN-SoLo. Similarly, for the liver dataset, POMS required 6,118 s, whereas ANN-SoLo completed the search in 4,127 s. Overall, POMS required approximately 1.5 times the runtime of the baseline ANN-SoLo method.

This additional runtime arises from the multi-representation search strategy, which independently probes four distinct spectral representations to maximize the recovery of shifted fragment-ion series. Despite the increased runtime, POMS provides substantially improved identification sensitivity, particularly for peptides containing challenging modifications such as those located near the C-terminus. Search performance is also influenced by the search depth within the Faiss-based ANN index (i.e., the number of probed clusters). Increasing the search depth generally improves identification sensitivity but proportionally increases computational time. In this study, the search parameters were selected to maintain a practical balance between identification performance and runtime. Under these conditions, POMS consistently identified more modified peptide–spectrum matches while maintaining search times that remain practical for large-scale proteomic datasets.

## Conclusions

Open modification search has become an essential strategy for exploring the diversity of PTMs in large-scale proteomic datasets. Spectral library searching approaches such as ANN-SoLo have significantly improved the scalability of OMS by leveraging ANN indexing to efficiently retrieve candidate spectra. Despite this progress, a fundamental limitation remains: ANN-based candidate retrieval relies on spectral vector similarity that does not explicitly account for modification-induced fragment-ion shifts. As a result, modified spectra that are otherwise highly similar to their unmodified counterparts can be excluded during the candidate selection stage.

In this study, we addressed this limitation by introducing POMS, a modification-aware candidate retrieval framework that integrates complementary spectral representations with sequence-derived theoretical fragment features. The central idea of POMS is to preserve peptide backbone similarity in vector space even when fragment ions are systematically shifted by modifications. By compensating for modification-induced spectral distortion, POMS improves the alignment between the vector similarity used for candidate retrieval and the spectral similarity evaluated during final scoring with the shifted dot product. In particular, whereas ANN-SoLo exhibits decreasing correlation between these metrics as modifications occur closer to the C-terminus, POMS maintains strong correspondence between vector similarity and spectral scoring. This improved alignment enables POMS to retrieve correct candidate spectra that would otherwise be excluded during ANN-based indexing. Benchmarking across two large-scale human MS/MS datasets demonstrated that this modification-aware retrieval strategy significantly improves the identification of modified peptides.

Beyond algorithmic improvements, the enhanced recovery of modified peptides achieved by POMS has important implications for proteomic analysis. Open modification searches are increasingly used to uncover unexpected or previously uncharacterized post-translational modifications in large-scale datasets. By improving the sensitivity of candidate retrieval—particularly for modifications located in structurally challenging regions such as the peptide C-terminus—POMS expands the detectable modification landscape and facilitates more comprehensive exploration of the proteomic modification space.

Despite these advances, several challenges remain. First, the current complementary transformation considers only singly charged fragment ions. Although POMS consistently outperformed ANN-SoLo across all precursor charge states examined, including higher-charge precursors (Supplementary Fig. S5), multiply charged fragment ions may become increasingly relevant for spectra from higher-charge precursors and are not explicitly represented in the current framework. Extending the complementary transformation to account for multiple fragment charge states, potentially in combination with robust charge-state assignment or spectral deconvolution, may further improve candidate retrieval for such spectra. POMS also showed improved identification of peptides carrying multiple phosphorylation sites (Supplementary Fig. S2), suggesting that the current representation remains effective for heavily modified peptides. Nevertheless, more complex modification patterns involving multiple co-occurring PTMs may benefit from further extensions of the spectral representation. Second, algorithmic trade-offs, such as the reduced ANN search space ratio required to maintain computational efficiency for the expanded index architecture, can occasionally limit sensitivity for certain N-terminal modifications. Future work will explore strategies to mitigate this limitation while further improving the scalability of ANN-based indexing for increasingly large proteomic datasets.

## Supplementary Information

Below is the link to the electronic supplementary material.

Supplementary Material 1

## Data Availability

The datasets analyzed in this study were obtained from the ProteomeXchange Consortium via the PRIDE repository under the dataset identifiers PXD001468, PXD000561, and PXD004452.

## Abbreviations

PTM: post-translational modification
FDR: false discovery rate
MS: mass spectrometry
MS/MS: tandem mass spectrometry
PSM: peptide-spectrum match
HEK: human embryonic kidney
ANN: approximate nearest neighbor
OMS: open modification search
