# Supplementary material for: POMS enhances open spectral library search for identification of modified peptides
Source: BMC Bioinformatics. 2026 Sep 21;27:265. doi: 10.1186/s12859-026-06672-0 (PMC13741594; doi:10.1186/s12859-026-06672-0)
Supplement: Supplementary file 1 [file 12859_2026_6672_MOESM1_ESM.pdf]

Supplementary Information

POMS enhances open spectral library search for identification of modified peptides

Younghee Seo[1], Eunok Paek[2,3], and Seungjin Na[1,4]**

[1]Center for Target-to-Therapeutics Research, Korea Basic Science Institute, Cheongju 28119, Republic of Korea,
[2]Department of Computer Science, Hanyang University, Seoul 04763, Republic of Korea,
[3]Department of Artificial Intelligence, Hanyang University, Seoul 04763, Republic of Korea,
[4]Convergent Analytical Science, University of Science and Technology, Daejeon 34113, Republic of Korea

Table of Contents

Table S1. Ablation study of POMS spectral representation features during the open search phase.

Table S2. ANN index architectures and search parameters used in ANN-SoLo and POMS.

Figure S1. Representative spectra illustrating how different spectral representations facilitate candidate retrieval depending on modification location.

Figure S2. Performance in a phosphopeptide-enriched dataset.

Figure S3. Global overlap of PSMs identified by ANN-SoLo, POMS, pFind3, and MSFragger.

Figure S4. Top-*k* candidate retrieval recall for modified peptides.

Figure S5. Identification performance stratified by precursor charge states during the open search phase.

Model	Liver Open Search (PSMs)	HEK293 Open Search (PSMs)
ANN-SoLo	67,418	284,401
Ablation 1 (Complementary Only)	70,972	293,278
Ablation 2 (Sequence Embedding Only)	72,299	289,706
POMS (Integrated)	72,862	290,670

Supplementary Table S1. Ablation study of POMS spectral representation features during the open search phase. The table presents the numbers of modified PSMs identified by the baseline ANN-SoLo, single-feature models (complementary peaks only and sequence embedding only), and the integrated POMS model across the Liver and HEK293 datasets. PSMs consistently identified during the initial standard search phase (217,536 for Liver and 534,576 for HEK293) were excluded to specifically evaluate candidate retrieval performance during the open search phase. While the relative contribution of each individual feature varied between datasets (e.g., sequence embedding for Liver and complementary peaks for HEK293), integrating both features in POMS provided robust improvements across both datasets.

Model	Vector Dimensionality	Sub-indices	nlist	nprobe	Search Ratio	Total Index Size
ANN-SoLo	800	1 (original)	256	128	50.0%	13 GB
Ablation 1 (Comp Only)	800	4	4,096	512	12.5%	51 GB
Ablation 2 (Seq Only)	1,600	1 (original)	256	128	50.0%	101 GB
POMS	1,600	4	4,096	512	12.5%	102 GB

Supplementary Table S2. ANN index architectures and search parameters used in ANN-SoLo and POMS. The table summarizes the vector dimensionality, index structure, and Faiss search parameters (nlist and nprobe) for each method. To accommodate the expanded 1,600-dimensional concatenated vector space and four parallel sub-indices in POMS, the search space ratio (nprobe / nlist) was adjusted to 12.5% to maintain practical computational runtimes, yielding a total index size of 102 GB. All methods retrieved a maximum of 1,024 candidates per query prior to the fine-scoring phase.

Supplementary Figure S1

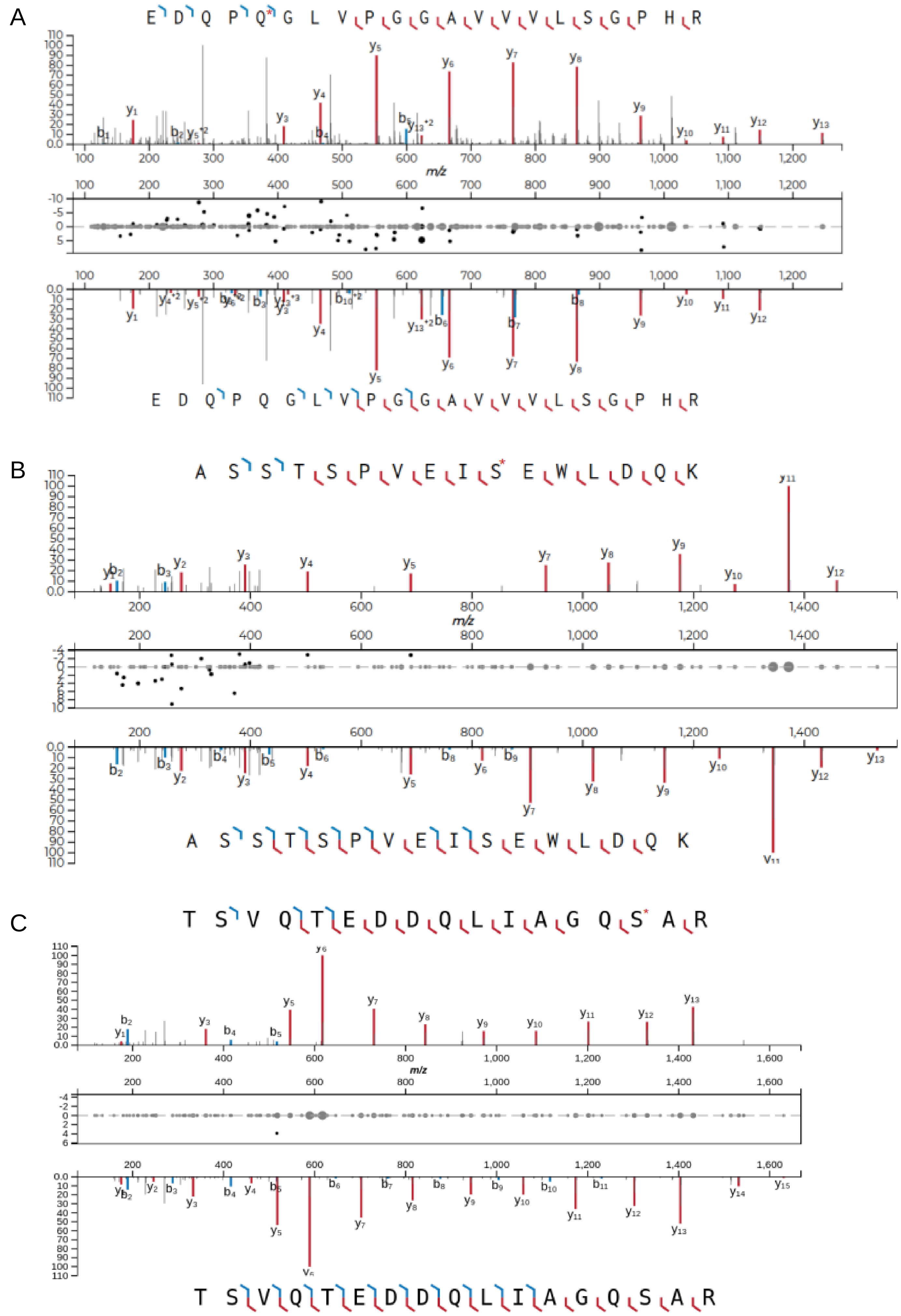

Supplementary Figure S1. Representative spectra illustrating how different spectral representations facilitate candidate retrieval depending on modification location. (A) Example of a spectrum with an N-terminal modification identified using the standard spectral representation. The peptide contains Deamidation at Q5 near the N-terminus. Because most y-ions remain unshifted, the modified query spectrum maintains strong similarity to the unmodified library spectrum and can be retrieved using the conventional ANN-SoLo representation. (B) Example of a spectrum with an internal modification identified using the high-m/z down-weighted representation. The peptide contains Formylation at S10 located in the middle of the sequence. The modification introduces a partial mass shift in the y-ion series beginning at y7. Down-weighting high-m/z peaks reduces the penalty associated with mismatches in this region and improves candidate retrieval for spectra with internal modifications. (C) Example of a spectrum with a C-terminal modification recovered using the complementary spectral representation. The peptide contains Formylation at S15 near the C-terminus. Because the modification shifts most dominant y-ions, the query spectrum becomes dissimilar to the unmodified library spectrum under conventional spectral hashing. The complementary representation transforms shifted ions into complementary positions corresponding to unmodified fragment ions, restoring spectral similarity and enabling successful candidate retrieval.

Supplementary Figure S2

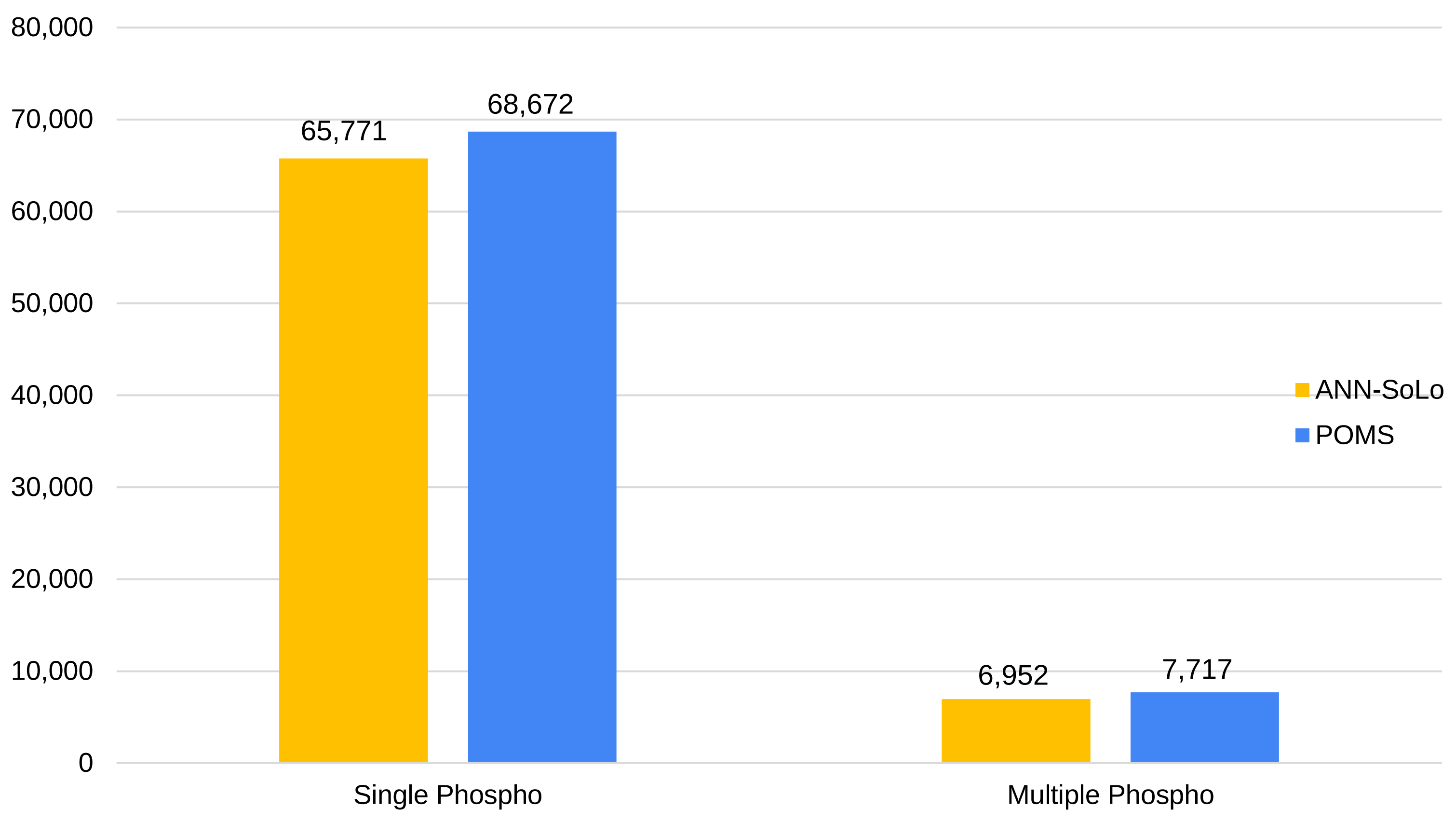

Supplementary Fig. S2. Performance in a phosphopeptide-enriched dataset. The bar chart compares the numbers of phosphorylated PSMs identified by ANN-SoLo and POMS in a phosphoproteomics dataset (PXD004452). Modification-associated mass shifts are categorized into single phosphorylation (~+80.0 Da) and multiple phosphorylation (~+160.0 and ~+240.0 Da, corresponding to double and triple phosphorylation, respectively). POMS improved the identification of phosphorylated peptides across these categories, with a greater relative improvement observed for multiply phosphorylated peptides, yielding an 11.0% increase compared with ANN-SoLo.

Supplementary Figure S3

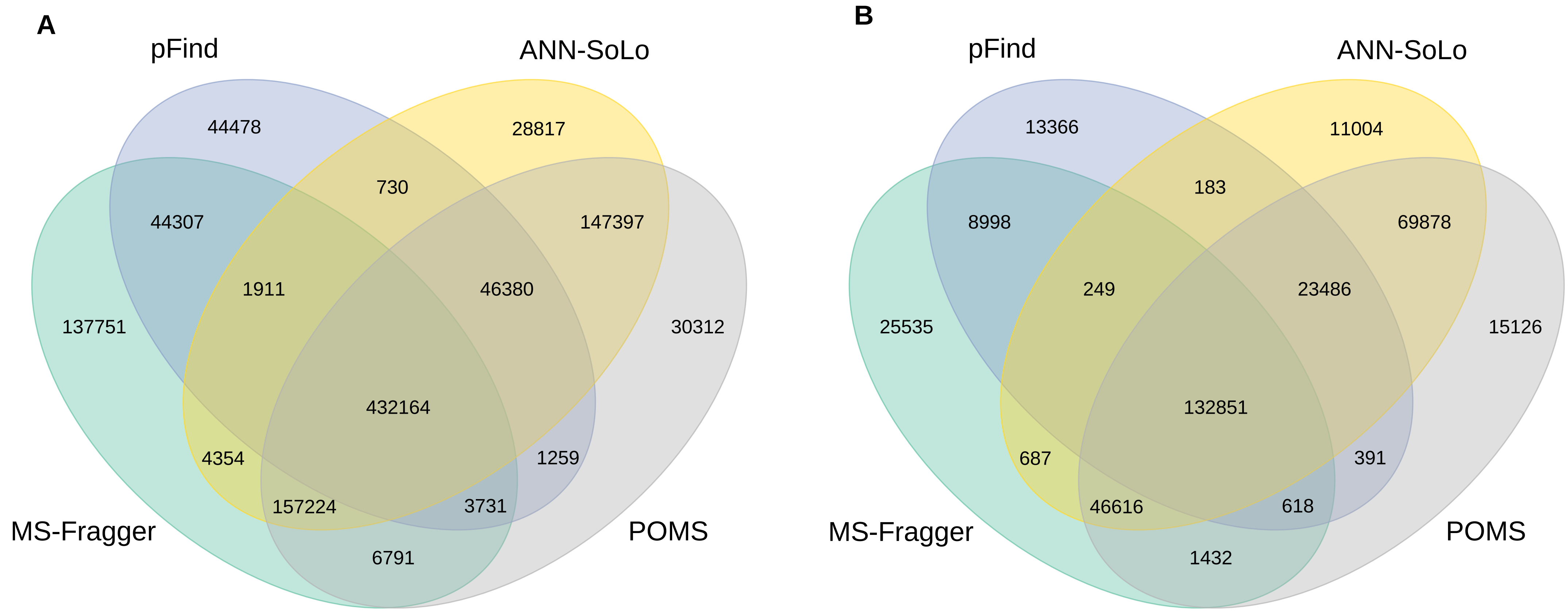

Supplementary Figure S3. Global overlap of PSMs identified by ANN-SoLo, POMS, pFind3, and MSFragger. Venn diagrams show the overlap of all identified PSMs across the four search methods for (A) the HEK293 dataset and (B) the human liver dataset. Overlap was defined when the same peptide sequence was assigned to the same query spectrum across methods.

Supplementary Figure S4

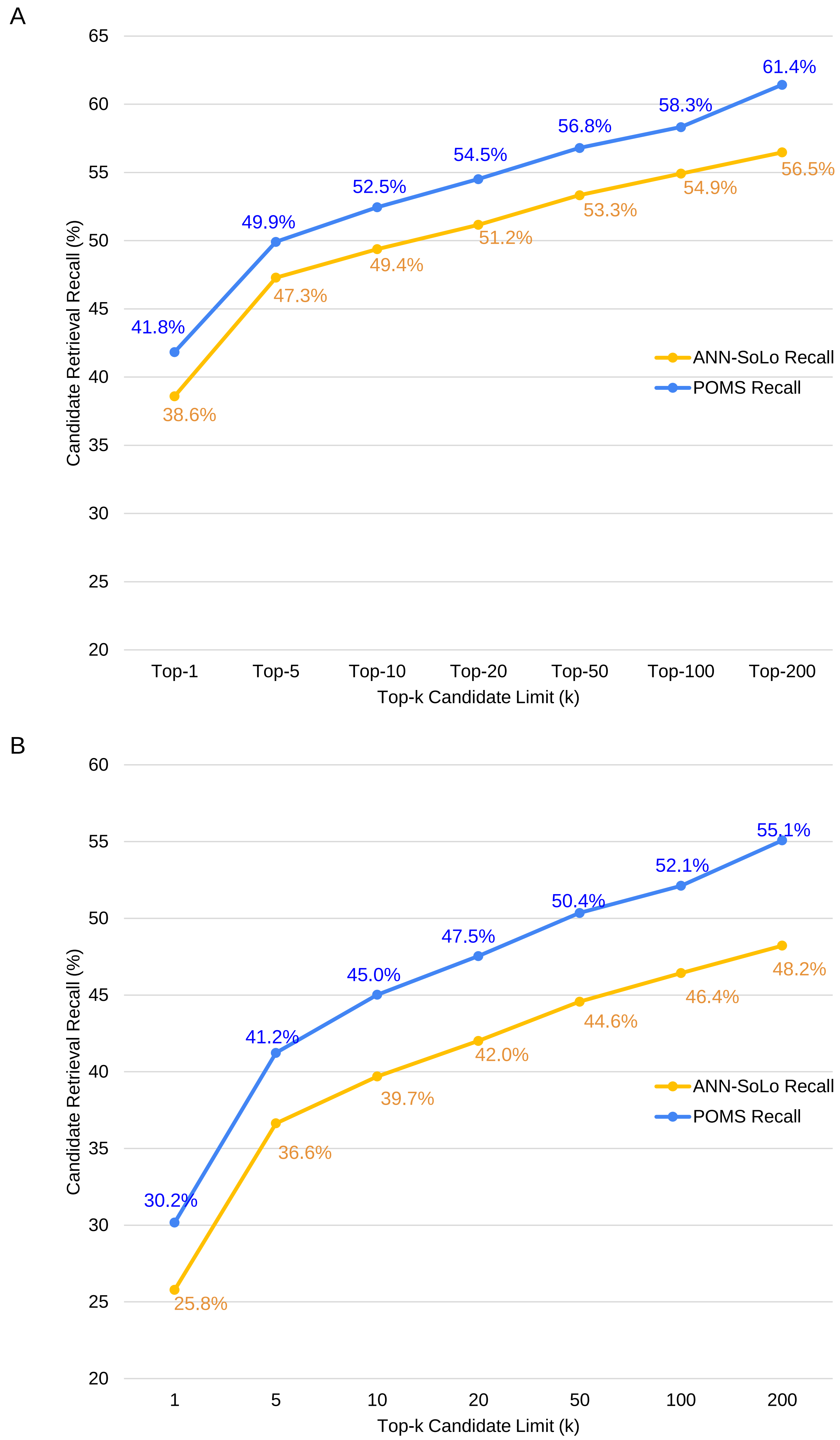

Supplementary Figure S4. Top-*k* candidate retrieval recall for modified peptides. The line graph compares the Top-*k* candidate retrieval recall of ANN-SoLo and POMS using ground-truth PSMs identified from the (A) HEK293 and (B) human liver datasets by pFind. Top-*k* recall was calculated as the proportion of ground-truth PSMs for which the correct peptide was represented among the Top-*k* candidates retrieved by ANN-SoLo or POMS (k = 1–200). POMS consistently achieved higher recall than ANN-SoLo across all *k* thresholds, demonstrating that the modification-aware spectral representation increases the likelihood of retrieving the correct peptide during the candidate retrieval stage.

Supplementary Figure S5

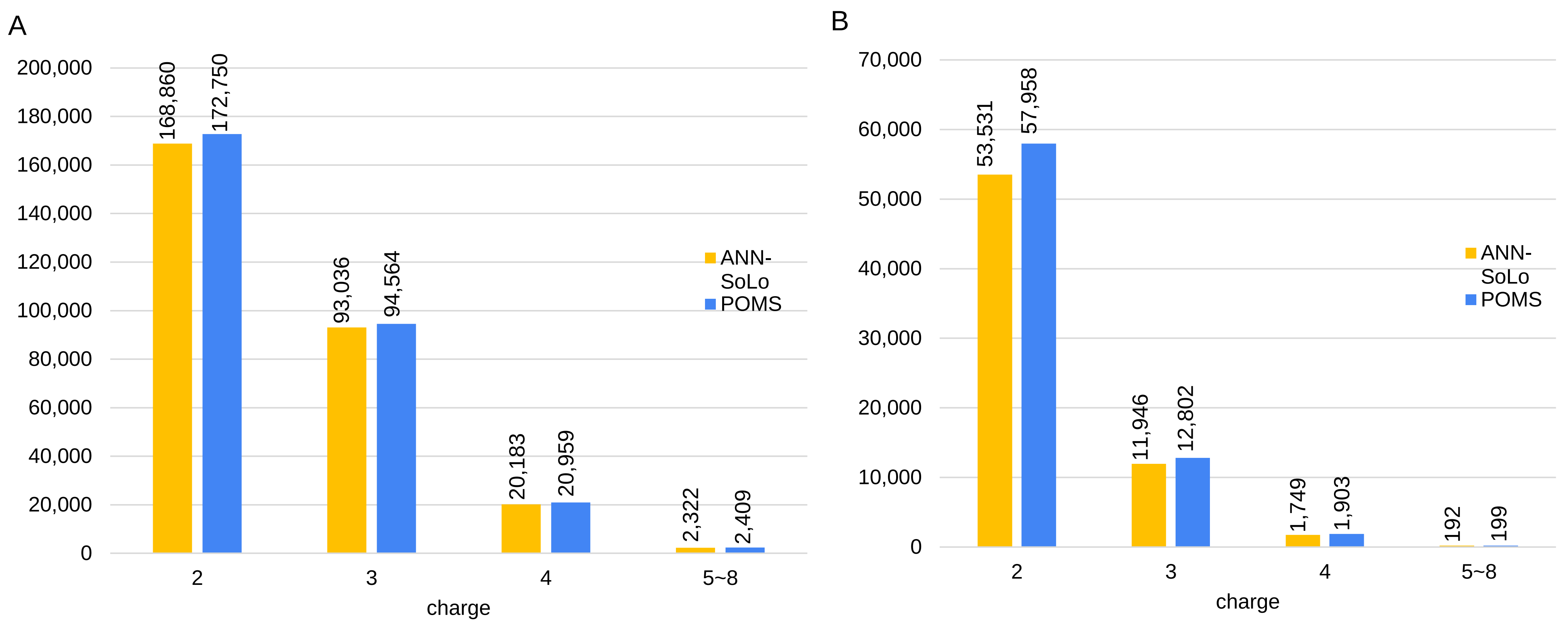

Supplementary Figure S5. Identification performance stratified by precursor charge states during the open search phase. The bar charts illustrate the numbers of modified PSMs identified by ANN-SoLo and POMS across precursor charge states (+2, +3, +4, and ≥+5) for the (A) HEK293 and (B) human liver datasets. PSMs identified during the initial standard search phase were excluded to specifically evaluate the identification performance for modified peptides during the open search phase. POMS consistently outperformed ANN-SoLo across all precursor charge states.
